# Social Interactions Are Related to Cognitive Development in Western Australian Magpie Fledglings

**DOI:** 10.1002/ece3.72435

**Published:** 2025-11-11

**Authors:** Elizabeth M. Speechley, Benjamin J. Ashton, Alex Thornton, Stephanie L. King, Leigh W. Simmons, Amanda R. Ridley

**Affiliations:** ^1^ Centre for Evolutionary Biology, School of Biological Sciences University of Western Australia Crawley Western Australia Australia; ^2^ College of Science and Engineering Flinders University Adelaide South Australia Australia; ^3^ Centre for Ecology and Conservation University of Exeter Cornwall UK; ^4^ School of Biological Sciences University of Bristol Bristol UK

**Keywords:** aggression, cognition, ontogeny, social network, vocal

## Abstract

Social interactions during development can have a significant and lasting impact on adult phenotypes and fitness. Indeed, a growing body of evidence suggests the early social environment plays an important role in cognitive development. However, existing studies largely focus on the impact of social group size, which does not necessarily capture all the cognitive demands associated with group living. Social network analysis can provide detailed insight into variation in social interactions between group members, and thus the information‐processing challenges associated with group living at the individual level. Here, we explore whether social interactions during development are related to cognitive performance in juvenile Western Australian magpies ( *Gymnorhina tibicen dorsalis* ). Specifically, we investigated the relationship between social network measures of connectedness (physical proximity, play, agonistic and vocal interactions) and individual cognitive performance, tested at three developmentally sensitive time points during the first year of life. We found that social measures were related to cognitive performance: individuals in larger groups solve an associative learning task in fewer trials at 300 days post‐fledging. Additionally, individuals that responded to vocalisations from more conspecifics and those that received aggressive interactions from more conspecifics perform better at an associative learning task at 300 days post‐fledging. Our study highlights the value of considering individual‐based social network measures, which capture the differences in specific social connections between individuals within groups, when investigating the relationship between the social environment and cognitive development.

## Graphical abstract

The early social environment can play a pivotal role in an individual's ability to learn, solve problems and make decisions. We find that multiple elements of an individual's social upbringing influence their ability to learn.

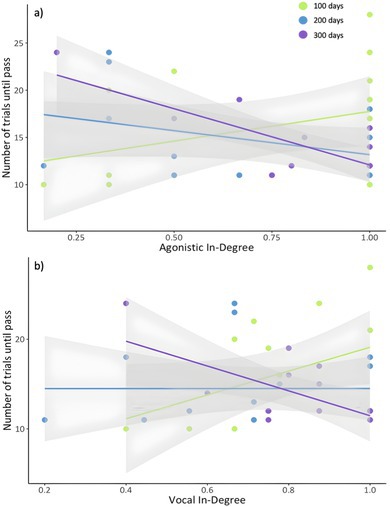

## 1 Introduction

Environmental influences during development can have a significant and lasting impact on adult phenotype and fitness (West‐Eberhard 2003). Rearing environments during the juvenile period can impact personality (Guenther et al. 2014), immune systems (Lubach et al. 1995), mating preferences (Riebel et al. 2009), courtship (Ruploh et al. 2013) and behaviour (Carducci and Jakob 2000). However, less is known about the influence of the rearing environment on cognitive development. The social environment in particular may influence the development of cognitive phenotypes during adolescence (Sachser et al. 2018). Cognition is defined as the mental processes by which animals collect, retain and use information from their environment to guide their behaviour and can vary widely between individuals and populations (Shettleworth 2001). The Social Intelligence Hypothesis (SIH) suggests that the demands of living in complex social groups are key drivers of cognitive evolution (Chance and Mead 1953; Dunbar 1998; Humphrey 1976; Jolly 1966, reviewed in Speechley, Ashton, Foo, et al. 2024). Maintaining and coordinating multiple relationships, observing and keeping track of group members, and recognising suitable cooperative partners, are a few of the many factors unique to social animals that are hypothesised to require a high degree of informational processing (Byrne and Whiten 1988; Chance and Mead 1953; Dunbar 1998; Humphrey 1976; Jolly 1966). Although the SIH was conceived as an evolutionary hypothesis, the underlying logic can also apply to developmental processes. For instance, social pressures during early life can drive individual cognitive development (Thornton and Lukas 2012). Consequently, the recent shift toward an intraspecific approach to the study of cognition, focusing on the causes and consequences of individual differences, has encouraged investigations of the SIH in a developmental context (Ashton, Thornton, and Ridley 2018; Thornton and Lukas 2012).

Developmental tests of the relationship between sociality and cognition repeatedly quantify cognition over multiple time points and gather longitudinal data to determine the factors affecting changes in cognitive phenotypes over time (Ashton, Thornton, and Ridley 2018). There is growing evidence that the early social environment can affect brain development and cognitive performance across a diverse range of vertebrates (Ash et al. 2020; Ferreira et al. 2025; Fischer et al. 2015; Munch et al. 2018; Speechley, Ashton, Thornton, Simmons, et al. 2024; Szabo and Ringler 2025; Triki et al. 2024). For example, Western Australian magpie fledglings ( *Gymnorhina tibicen dorsalis* ) raised in larger groups performed better in a cognitive test battery than those in smaller groups (Ashton, Ridley, et al. 2018). Additionally, in the daffodil cichlid ( *Neolamprologus pulcher* ), rearing group size influenced the development of social skills and the relative size of several brain regions (Fischer et al. 2015). However, a number of studies have reported findings inconsistent with the idea that the social environment has an influence on cognitive development (Riley et al. 2017, 2018; Soravia et al. 2022)—for example, no relationship was found between social rearing conditions and learning performance in tree skinks (*Egernia striolata*) (Riley et al. 2017).

Developmental studies of the relationship between sociality and cognition have largely focused on the impact of social group size on cognitive development. In these studies, individuals have either been raised in isolation (Ausas et al. 2019), raised without parents (Enthoven et al. 2008), or raised in different‐sized groups (Taborsky et al. 2012). Although group size provides a useful measure of the largest potential number of conspecifics with which an individual can interact, it is unlikely that all individuals within a social group will be interacting equally (Morrison et al. 2020). Furthermore, interacting with more individuals does not necessarily entail greater information‐processing challenges, as animals may not necessarily discriminate between or respond differentially toward different conspecifics (Morrison et al. 2020). Certain types of social interaction may also influence specific cognitive traits. For example, a study of captive common marmosets ( *Callithrix jacchus* ) found that juveniles who frequently vocalised with conspecifics made fewer learning errors (Ash et al. 2020). However, they found no significant relationship between learning and group size, proximity or grooming (Ash et al. 2020). Therefore, it is crucial to explore individual‐based measures of cognition, as well as individual‐based measures of sociality, to determine exactly which social pressures are influencing which aspects of cognitive development.

Highly social species that readily interact with cognitive tests provide ideal models to explore which social pressures influence cognitive development. Here, we test whether social connectedness during early development influences cognitive development in social, cooperatively breeding, wild Western Australian magpies (a species known to readily interact with cognitive tasks, Ashton, Ridley, et al. 2018; Speechley, Ashton, Thornton, et al. 2024). Specifically, we investigate several network types designed to quantify social connectedness (including play, agonistic and vocal interactions, and physical proximity), and their relationship with associative learning performance (a domain‐general cognitive trait that can be used in diverse social contexts (Morand‐Ferron 2017)) tested at three time points during the first year of life. Play, vocal communication and agonistic interactions, as well as physical proximity are commonly used to quantify social connectedness and presumed to capture multiple aspects of social life, including affiliation, conflict and social tolerance (Croft et al. 2008). Given that fledglings raised in larger groups were previously shown to perform better in cognitive tests at 200 and 300 days post‐fledging (Ashton, Ridley, et al. 2018), we predict that individuals who are involved in more interactions (“strength” in social network terms, Table 1), with more individuals (“degree”, Table 1) will perform better in an associative learning task. We also predict that where these interactions are more differentiated (“coefficient of variation”, Table 1), individuals will perform better in associative learning due to the informational demands of remembering, recognising and responding appropriately to conspecifics by optimising their social behaviour to the available social information.

**TABLE 1:** Definitions of predictor variables used in analysis.

Glossary of terms	
Affiliative degree	Number of conspecifics the individual participates in affiliative interactions with (normalised for group size, values ranging from 0 to 1)
Affiliative strength	Frequency of affiliative interactions an individual participates in (normalised for group size, values ranging from 0 to 1)
Age	Age tested: corresponding to approximately 100, 200 or 300 days post‐fledging
Agonistic degree	Number of conspecifics the individual interacts with aggressively (normalised for group size, values ranging from 0 to 1)
Agonistic in‐degree	Number of conspecifics the individual receives aggressive interactions from (normalised for group size, values ranging from 0 to 1)
Agonistic out‐degree	Number of conspecifics the individual initiates aggressive interactions toward (normalised for group size, values ranging from 0 to 1)
Agonistic strength	Frequency of aggressive interactions an individual participates in (normalised for group size, values ranging from 0 to 1)
Agonistic in‐strength	Frequency of aggressive interactions an individual receives (normalised for group size, values ranging from 0 to 1)
Agonistic out‐strength	Frequency of aggressive interactions an individual initiates (normalised for group size, values ranging from 0 to 1)
Agonistic COV	Coefficient of variation (COV) is the number of differentiated relationships or interactions within the agonistic network
Body mass	Individual body mass collected within a week of testing to 0.1 g accuracy (averaged where multiple measures available for an individual)
Brood mates	Presence or absence of fledglings from the same nest
Foraging efficiency	Amount of food (grams) caught per minute foraging. Based on focal observations conducted within a week of testing (averaged where multiple measures available for an individual)
Group size	Number of adult individuals (> 2 years) in the group at time of testing
Neophobia	Time taken to approach the test apparatus from 1 m (seconds)
Number of fledglings	The number of fledglings present in the group at the time of testing
Order tested	Order the individual was tested in each group
Shade	Rewarded shade of the associative learning array (i.e., light or dark)
Vocal degree	Number of conspecifics the individual interacts with vocally (normalised for group size, values ranging from 0 to 1)
Vocal in‐degree	Number of conspecifics the individual receives vocal interactions from (normalised for group size, values ranging from 0 to 1)
Vocal out‐degree	Number of conspecifics the individual initiates vocal interactions toward (normalised for group size, values ranging from 0 to 1)
Vocal strength	Frequency of vocal interactions an individual participates in (normalised for group size, values ranging from 0 to 1)
Vocal in‐strength	Frequency of vocal interactions an individual joins (normalised for group size, values ranging from 0 to 1)
Vocal out‐strength	Frequency of vocal interactions an individual initiates (normalised for group size, values ranging from 0 to 1)
Vocal COV	Coefficient of variation (COV) is the number of differentiated relationships or interactions within the vocal network

## 2 Methods

### 2.1 Study Species and Site

Western Australian magpies are large passerines (250–400 g), living in highly territorial groups varying in size between 3 and 12 adults (Edwards et al. 2015; Pike et al. 2019) that remain relatively stable over time (Ashton, Ridley, et al. 2018). Groups in our study population are located in urban territories of open grassland and parkland in Guildford (31°89′S, 115°96′ E) and Crawley (31°98′S, 115°81′ E), Western Australia (Ashton, Ridley, et al. 2018). During data collection, our study population consisted of 80–120 individuals across 18 groups, with an average of 5.59 ± 0.60 adults and 1.54 ± 0.30 fledglings per group.

The study population has been monitored regularly since 2014. Individuals are habituated to observers, which facilitates close behavioural observation and presentation of cognitive tasks within 5 m (Ashton, Ridley, et al. 2018). Most individuals are individually identifiable via coloured metal rings, or through recognisable scarring or plumage aberrations. Males and females are sexually dimorphic, and thus sex is unknown until adult plumage is reached at 2–3 years of age (Ashton, Ridley, et al. 2018).

The breeding season begins in August and lasts until early summer (Pike et al. 2019). Magpies are facultative cooperative breeders (Pike et al. 2019). Most adults will attempt to breed, but only some receive help from other group members (Pike et al. 2019). Females are responsible for incubation, which lasts approximately 3–4 weeks (Pike et al. 2019). Once chicks have hatched, they are fed by the mother and may be aided by the father and helpers which may be male, female or juveniles (Pike et al. 2019). Magpie chicks spend approximately 3–4 weeks in the nest before fledging (Pike et al. 2019). Nests are monitored daily during the breeding season to determine an accurate date of fledging for each individual. Once fledged, they remain dependent on adults for food for an extended period (Pike et al. 2019). Young typically remain in their natal territory throughout their life, with dispersal rarely observed (< 1 dispersal/year) over the 11 years of the research project.

### 2.2 Associative Learning

Fledgling cognitive performance was tested in an associative learning task. Associative learning is likely to be highly ecologically relevant because it allows animals to make predictable associations between cues in the environment, such as associating the posture of a conspecific with incoming aggression (Morand‐Ferron 2017; Morand‐Ferron et al. 2016). Repeated cognitive testing has been validated in this species, with previous research finding that cognitive performance does not improve with repeated testing via causally identical, visually distinct tasks (Sollis et al. 2022). Previous studies have also repeatedly shown high levels of individual repeatability in adult magpies (Ashton, Ridley, et al. 2018; Ashton, Thornton, Cauchoix, et al. 2022; Speechley, Ashton, Thornton, et al. 2024) and that cognitive performance does not differ between ringed and unringed individuals (Ashton, Thornton, Speechley, et al. 2022).

Fledglings were tested at three time periods during their first year of life: 100, 200 and 300 days post‐fledging, following the procedure established by Ashton, Ridley, et al. (2018). The testing time periods correspond to developmental milestones: individuals start foraging independently at ~100 days post‐fledging and thus can readily engage with the cognitive task (Ashton, Ridley, et al. 2018). 200 days post‐fledging corresponds to when most fledglings stop being provisioned by other group members (Pike et al. 2019), and 300 days post‐fledging corresponds to the transition into the juvenile stage of development (Ashton, Ridley, et al. 2018). Cognitive testing was conducted in 2020 and 2021 between January and March (100 days post‐fledging), April and June (200 days post‐fledging) and August and September (300 days post‐fledging) at randomised times between 5:00 a.m. and 11:00 a.m. We attempted to test all fledglings within a group at all testing periods. However, we were unable to test some individuals if they were unable to be clearly distinguished from other fledglings, if they were too neophobic to interact with the task, or if they died during the testing period (see Table S2 for total numbers). Individuals were tested until they either passed the task or stopped engaging with the task (indicative of satiation). If individuals were unable to complete the task within a day due to satiation, loss of motivation or repeated failures, testing continued over consecutive days until the individual passed (74.68% passed within 1 day, 20.25% within 2 days and the remaining 5.07% in 3–4 days. All individuals who began the task passed within 4 days). Trials were conducted in social isolation (with conspecifics at least 10 m away), which minimised the potentially confounding factors of social learning or interference on cognitive performance (Ashton, Ridley, et al. 2018). As magpies frequently forage 10–20 m apart, we were able to ensure isolation for the duration of each trial.

**FIGURE 1:**
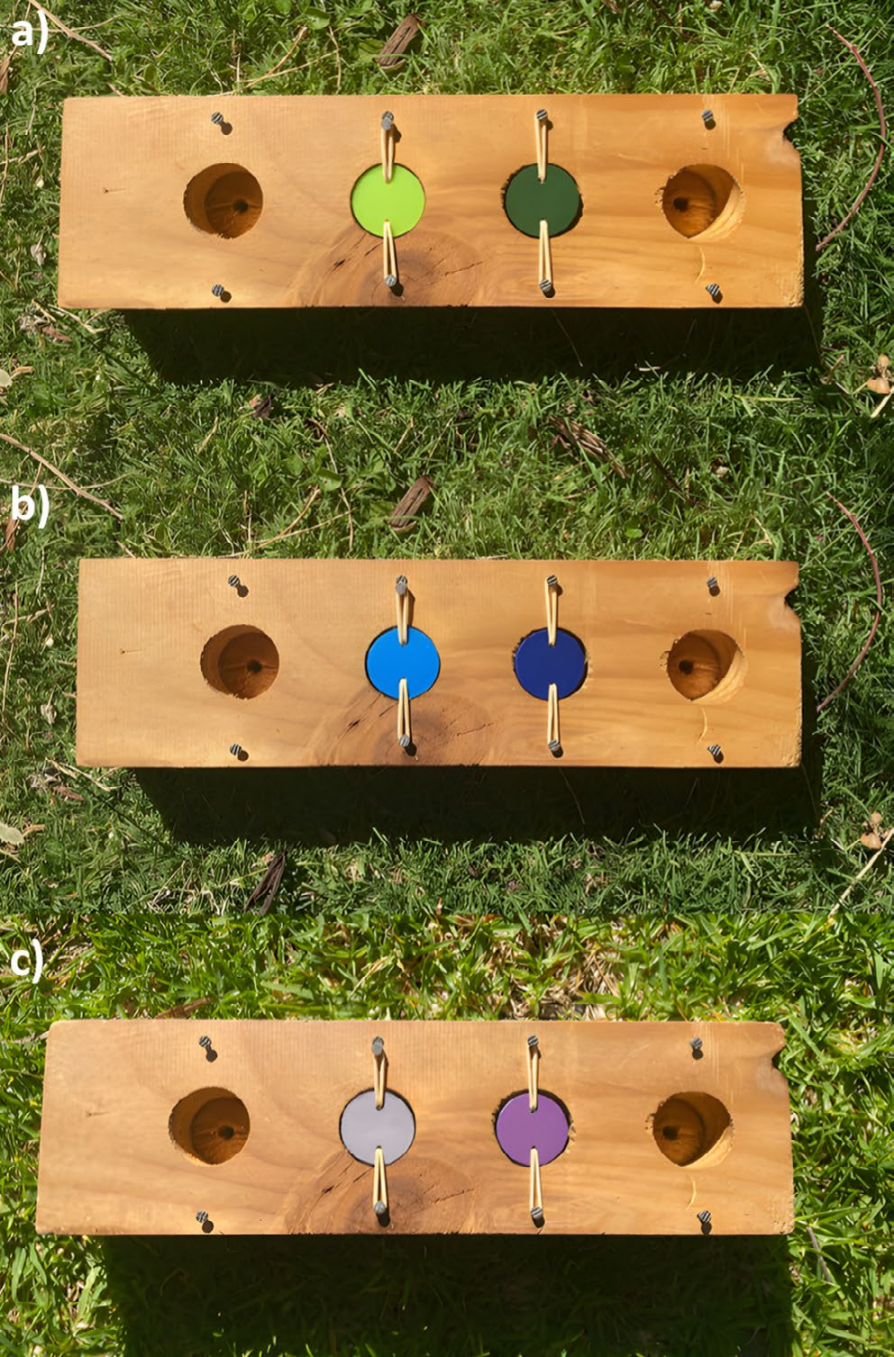
Associative learning array with colour combinations presented to fledglings at (a) 100 days, (b) 200 days and (c) 300 days post‐fledging. All individuals received the same colour combination at each testing period.

The associative learning array contained two equidistant wells (3.2 cm diameter × 1.5 cm deep, 3.5 cm apart) in a wooden grid (31 cm × 9 cm × 4 cm). PVC lids painted with two different shades of a single colour covered the wells. Lids were painted two different shades of colour, rather than two distinct colours to limit pre‐existing colour‐biases influencing performance (Boogert et al. 2018; Rowe and Healy 2014). Lids were fastened to the sides of the well by elastic bands threaded through drilled holes in the lids, which allowed the lids to swivel when pecked. The arrays for each testing period were causally identical, but visually distinct: individuals were presented with a unique colour combination at each time period (Figure 1). Before testing, both wells were rubbed with cheese to reduce the possibility that olfactory cues could be used to locate the rewarded well. For each individual tested, one shade was randomly selected as the rewarded shade for the duration of the testing period (the time taken to pass the task for that developmental stage). For each trial, the task was placed 5 m in front of the individual. During the initial training phase, the test subject was allowed to search both wells to see that only one well contained a reward. However, in all subsequent testing trials, they were only allowed to search one well before the apparatus was removed. The test subject had a minimum one‐minute interval between trials. An individual was considered to have passed the task when they selected the rewarded shade in 10 out of 12 consecutive trials (representing a significant deviation from random binomial probability, Ashton, Ridley, et al. 2018). Trials did not commence if the individual was unmotivated or stopped engaging with the task. We also pseudo‐randomised the side of the board that the rewarded well was located on so that it was not on the same side of the array for more than three consecutive trials. This ensured that the association was made between the shade and reward, not the orientation on the board.

### 2.3 Additional Explanatory Factors

We recorded several other factors that may potentially influence cognitive performance (Table 1) (Shaw and Schmelz 2017; Shaw 2017), including the shade of the rewarded well (light or dark), neophobia (defined as the time elapsed (seconds) from approaching the test apparatus from 1 m away and first making contact with the apparatus), body mass (grams, see below), foraging efficiency (amount of food caught in grams per minute foraging, see below) and the order tested within the group (to investigate any potential social learning). We measured average ambient temperature (°C) at the time of testing using data from the Bureau of Meteorology (Bureau of Meterology 2025) to ensure that temperature did not impact cognitive performance. All testing was conducted at temperatures below 30°C (mean temperature during testing at the 100 day milestone was 21.92°C ± 0.42°C, range: 14.7°C–30°C; 200 days = 12.81°C ± 0.51°C, range: 3.5°C–24.2°C; 300 days = 12.52°C ± 0.53°C, range: 5°C–19°C) as temperature above 32°C is known to affect cognitive performance in this species (Blackburn et al. 2022).

To account for differences in motivation, we also measured several proxies of body condition to ensure that hunger was not associated with cognitive performance (Rowe and Healy 2014). We collected data on body mass by encouraging individuals to hop onto a top‐pan scale (Ohaus Challenger series, 1000 ± 1 g) for a small food reward (< 1 g mozzarella cheese) (Edwards et al. 2015). We also conducted weekly ten‐minute foraging focal observations to estimate foraging efficiency. These observations were standardised to include active foraging during at least 50% (5 min) of the focal to ensure focals provided a representative sample of foraging efficiency. We used a customised program in Cybertracker (Liebenberg 2003) to record foraging focal observations and individual foraging efficiency was calculated as the amount of food (in grams) caught per minute of time spent foraging (Edwards et al. 2015). Both body mass measurements and foraging focals were collected during the morning (5:00 a.m. to 11:00 a.m.) within one week after completion of cognitive testing.

### 2.4 Social Networks

Social network data was collected according to the procedure outlined by Speechley, Ashton, Thornton, et al. 2024. Weekly social network observations were conducted from February to June 2020 and 2021 and August to October 2020 and 2021, with 10 observations per group per testing period (40 social network observations in total per group, except for one group, where *n* = 30). Network observations were conducted at these times to align with developmental time periods. Each observation was conducted over 90 min at varying times from 5:00 a.m. to 11:00 a.m. Interactions were recorded *ad libitum* on a Samsung Galaxy Tablet A6 using a free customisable, ethological software (Cybertracker) (Liebenberg 2003). We only conducted social network observations on groups where all individuals were identifiable. As magpies are a group‐living species that move together throughout the day, it was possible to keep track of all group members during an observation. However, recordings were paused if all individuals were not in sight and recommenced once all individuals were visible and accounted for.

A total of 765 h of social network observations were conducted (180 h on 12 groups from February to June 2020, 195 h on 13 groups from August to October 2020, February to June 2021 and August to October 2021). All‐occurrence sampling was used to record play, agonistic and vocal interactions. Agonistic interactions consisted of fighting, chasing, pecking, splays (a dominance display) and physical attacks. Vocal interactions were restricted to vocal choruses, as this was the only frequently occurring vocalisation with an identifiable initiator and receiver. The chorus is a common vocalisation in magpies that is given in a variety of contexts, including during competitive inter/intra‐group interactions, group disturbances and mating and social cohesion (Dutour and Ridley 2020; Speechley, Ashton, Thornton, et al. 2024). Where possible, we identified the actor (initiator) and receiver of all social interactions, as well as the frequency of interactions.

Proximity data were collected using scan sampling by recording the approximate distance (metres) between each individual in the group every 15 min. Group sampling was preferred to focal sampling as it maximises the number of potential interactions recorded during a single sampling event and has been shown to perform better than focal sampling methods for proximity data (Davis et al. 2018). To ensure that proximity was recorded accurately and consistently, two observers (E.M.S. and a volunteer) estimated distances ranging from 1 to 120 m (representing the range of distances recorded during the observations) with 89% and 88% accuracy, respectively.

### 2.5 Statistical Analysis

#### 2.5.1 Social Networks

All network analyses were conducted in R v.4.2.1 (R Core Team 2022). We analysed each group separately at each observation period (see above for observation period dates), with a separate network for each interaction type (play, agonistic, vocal and proximity). We converted proximity data into association matrices using the Simple Ratio Index (SRI) (Hoppitt and Farine 2018) via the *asnipe* package (Farine 2013). The SRI is an estimate of the proportion of time two animals spend together (0 for pairs of animals never observed together; 1 for pairs always seen together). We ran proximity networks at four different cut‐offs, restricting datasets to proximity measures within 1, 2, 5 and 15 m to determine an appropriate cut‐off for proximity networks. Since networks based on 1 m proximity showed the most variation in relationship strength (indicated by the highest coefficient of variation (COV) values and permutations significantly different to random), a proximity cut‐off of 1 m was used for all analyses (Table S1). Custom‐written R scripts were used to convert interaction data into directed network matrices (agonistic and vocal) or undirected network matrices (play). To confirm that the observed networks were significantly different to those expected by chance, permutations were run for each network (Farine 2013).

From these matrices, the *sna* package (Butts 2008) was used to calculate two node‐level network measures (degree and strength). Each of these metrics quantified a different aspect of social network centrality. Degree is the number of conspecifics the individual interacts with, whereas strength (also referred to as weighted degree centrality) is the sum of the weighted edges connected to an individual and represents how well‐connected an individual is in its network (Table 1) (Farine and Whitehead 2015). For directed networks (agonistic and vocal) which involve an actor and a receiver, in‐degree (e.g., the number of conspecifics the individual receives interactions from), out‐degree (the number of conspecifics the individual initiates interactions toward), in‐strength (the frequency of interactions the individual received) and out‐strength (the frequency of interactions the individual initiates) were also calculated (Table 1) (Farine and Whitehead 2015). We also calculated the coefficient of variation for each network, which represents the number of differentiated relationships or interactions within a network (smaller COVs indicate more homogeneous associations or interactions between group members, while higher COVs indicate more heterogeneous associations or interactions, Table 1). As groups vary in size, node‐level network measures were normalised to allow for comparison between groups. i.e., node strength for each individual was divided by the highest node strength within that group, thus scaling node strength between 0 and 1 (where individuals with a score of 1 showed the most social connectedness in that group). We also checked that our analyses were robust to multicollinearity between our social metrics (group size and social network metrics) using the variance inflation factor (with a VIF < 2 indicating low correlation).

Finally, to determine the minimum number of observations required for each individual in order for them to be included in the analysis, we conducted a sensitivity analysis. Based on this output, only individuals with a minimum of 10 focal observations were included in the final analysis (Figure S1), allowing a good balance between improved accuracy of network metrics and not excluding too many individuals.

#### 2.5.2 Predictors of Cognitive Performance

All statistical analyses were conducted in R v.4.2.1 (R Core Team 2022). The data were initially checked to confirm it met model assumptions, including the normality of residuals, presence of outliers and dispersion using the *DHARMa* package (Hartig 2021).

Before the main analysis, we ran a series of exploratory generalised linear mixed models (GLMMs) with a negative binomial distribution using the *lme4* package (Bates et al. 2015) with the combined scores for all ages. This exploratory analysis was designed to test potential factors that might confound cognitive performance, including the shade of the rewarded well, neophobia, body mass, foraging efficiency, order tested and average ambient temperature as explanatory terms. Fledgling associative learning score (number of trials taken to pass the task) was the response term and individual identity was included as a random term to account for repeated testing of the same individuals.

To test the hypothesis that the social environment affected the development of fledgling associative learning, we ran a series of GLMMs with a negative binomial distribution as described above. Offspring associative learning score (number of trials taken to pass the task) was the response term. To explore changes in performance with age, scores for all ages were combined to include all cognitive performance data, across all ages, in a single analysis and age was included as an explanatory factor. To explore the influence of the social environment we included the total number of fledglings in the group, the presence or absence of brood mates (fledglings from the same nest) and group size as explanatory factors. The total number of fledglings was included because fledglings spent a substantial amount of time playing with each other (play largely involved fledglings and juveniles, with only five instances of play recorded between a fledgling/juvenile and an adult), which may impact their cognitive development (Hill et al. 2017). Whereas group size was included as a proxy for social interaction with *all* group members. The presence or absence of brood mates was also included because fledglings may spend more time interacting with siblings, as opposed to other fledglings (non‐siblings) in the group. We also included an age by group size interaction to explore developmental changes in score at different group sizes. Individual identity was included as a random term to account for repeated testing of the same individuals (group identity could not be included as a random factor due to sample size constraints and explained less variation in the data than individual identity).

For the subset of individuals with social network measures, we ran another series of GLMMs with a negative binomial distribution. Offspring associative learning score (number of trials taken to pass the task) was the response term. To explore changes in performance with age, scores for all ages were combined to include all cognitive performance data, across all ages, in a single analysis and age was included as an explanatory factor. In this analysis, we used the explanatory factors listed above, as well as social network metrics including strength and coefficient of variation for the play networks; normalised in‐degree, out‐degree, in‐strength, out‐strength, and coefficients of variation for the agonistic and vocal networks (Table 1). Network metrics collected during February to June were paired with cognitive scores obtained during this time period (i.e., at 100 and 200 days post‐fledging), likewise network metrics collected during August to October were paired with cognitive scores collected during this time (i.e., 300 days post‐fledging). Individual identity was included as a random term to account for repeated testing of the same individuals (group identity could not be included as a random factor due to sample size constraints and explained less variation in the data than individual identity). Degree of play was removed from analyses due to lack of variation in the data (87% of individuals had a score of 1). Additionally, agonistic and vocal degree and strength were excluded as they correlated highly with either of the in/out degree or strength measures (VIF > 2), but explained less of the variation in the data than the term they were correlated with. Proximity metrics were omitted from analysis because 46 out of 51 networks were not significantly different from random (Table S3).

We determined the best model using a model selection process and terms were ranked in order of their corrected Akaike Information Criterion (AICc) score (AIC corrected for small sample sizes, Harrison et al. 2018). If a model was within two ΔAICc units of the top model with the lowest AICc and had 95% confidence intervals that did not intersect zero (Grueber et al. 2011), it was included in the top model set. Sample sizes reflect the datasets after removing missing values and all models tested in each model set had the same sample size.

## 3 Results

### 3.1 Associative Learning

We quantified associative learning performance in 29 individuals from 9 groups at 100 days post‐fledging, 27 individuals from 10 groups at 200 days post‐fledging, and 18 individuals from 8 groups at 300 days post‐fledging (all but 3 of the 18 individuals tested at 300 days were the same individuals tested at 100 days). The lower sample size at 300 days was due to fledgling mortality. 14 individuals completed the associative learning task at all three testing periods. The average number of trials to reach the passing criterion was 15.28 ± 0.95 at 100 days (range: 10–28 trials), 13.98 ± 0.68 at 200 days (range: 10–23) and 15.17 ± 1.54 at 300 days (range: 10–37; Figure 2).

**FIGURE 2:**
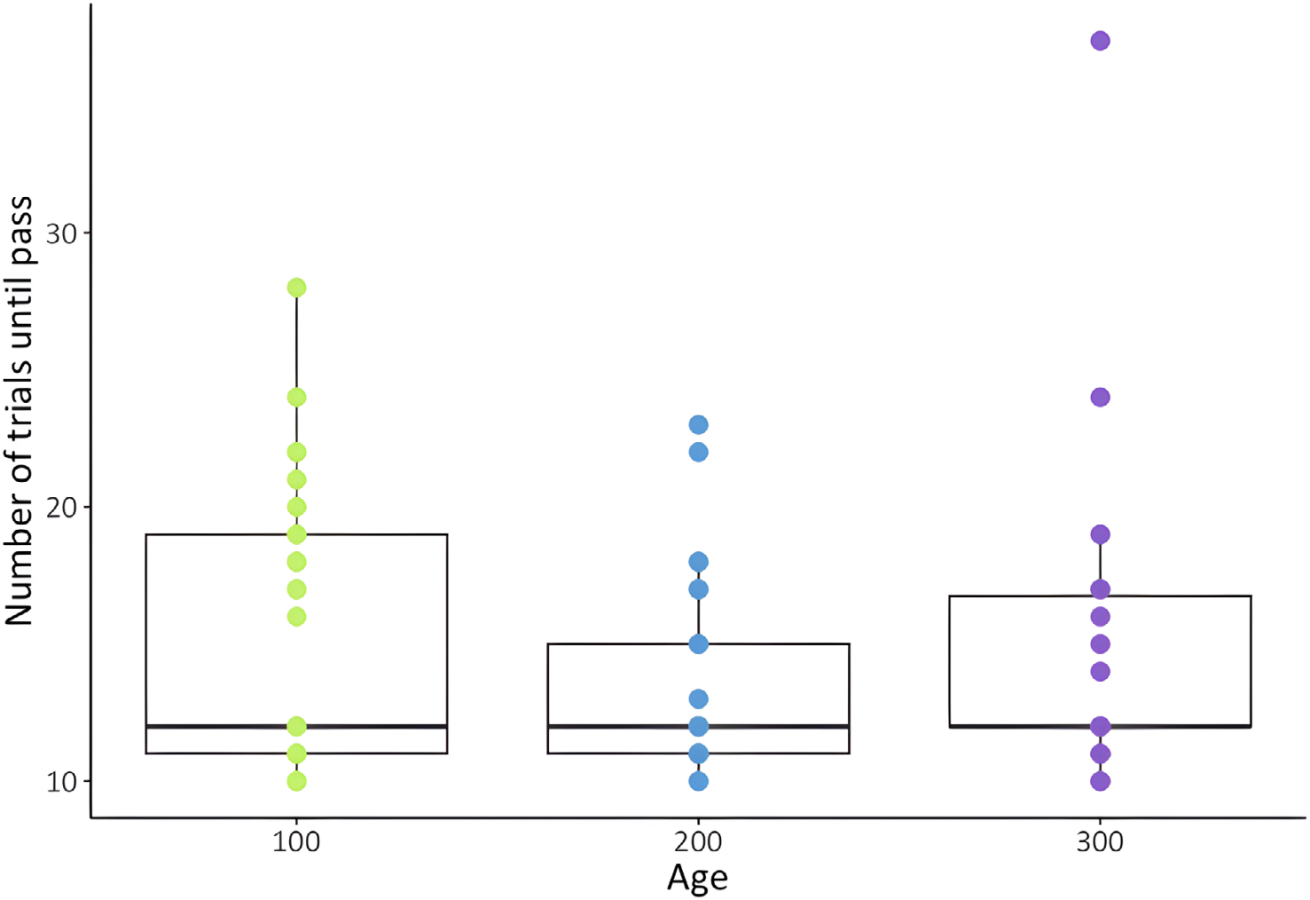
Boxplot of number of trials to pass the associative learning task at 100, 200 and 300 days post‐fledging (*N* = 29 individuals from 9 groups, 27 individuals from 10 groups, and 18 individuals from 8 groups, respectively). A lower number of trials indicates better performance in the task. The box represents 50% of the variation and the whiskers represent the 5th and 95th percentiles. The horizontal midline represents the median. Points represent individual scores.

### 3.2 Predictors of Cognitive Performance

**FIGURE 3:**
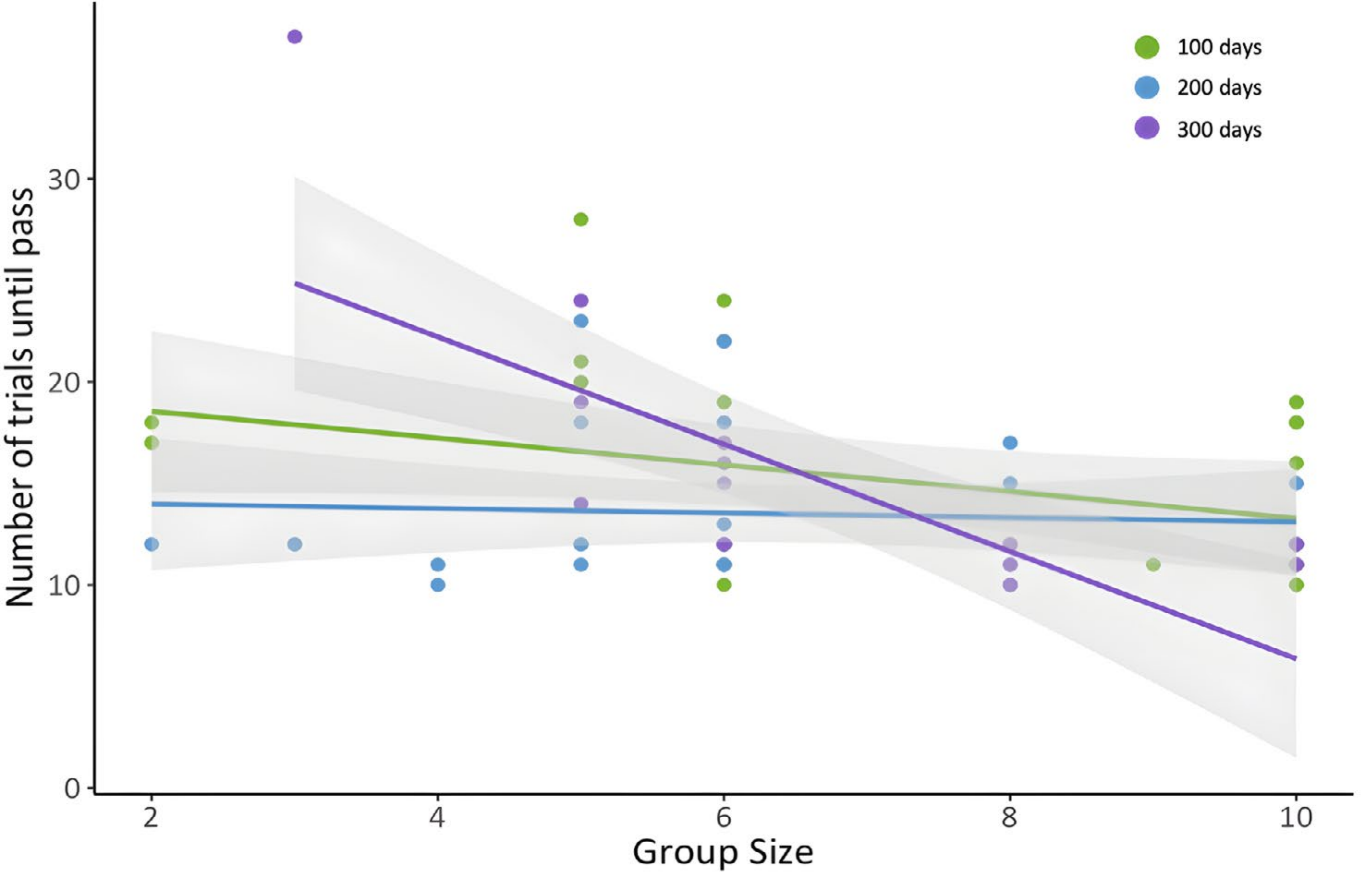
Relationship between group size and the number of trials taken to complete the associative learning task at each testing period (*N* = 74 cognitive tests on 34 individuals from 10 groups). Green points and line correspond to 100 days post‐fledging, blue represents 200 days post‐fledging and purple represents 300 days post‐fledging. A lower number of trials indicates better performance in the task. Points are raw data, the solid lines represent the line of best fit and the shaded areas represent the 95% confidence intervals.

**FIGURE 4:**
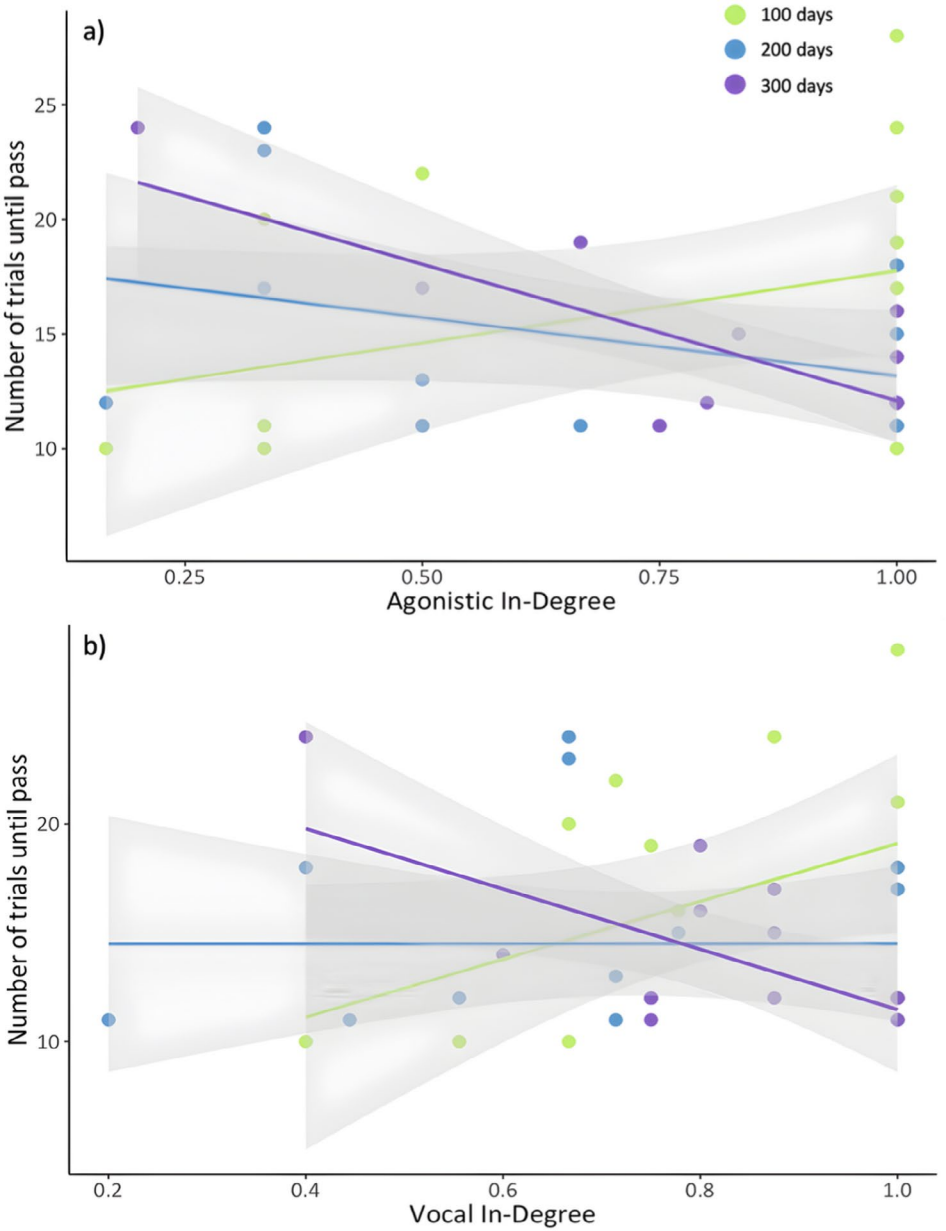
Relationship between the number of trials taken to complete the associative learning task and (a) agonistic in‐degree and (b) vocal in‐degree at each testing period (*N* = 46 cognitive tests on 21 individuals from 8 groups). Green points and line correspond to 100 days post‐fledging, blue represents 200 days post‐fledging and purple represents 300 days post‐fledging. A lower number of trials indicates better performance in the task. Points are raw data; the solid lines represent the line of best fit and the shaded areas represent the 95% confidence intervals.

Based on our exploratory analysis, cognitive performance was not related to any of our proxies of body condition/motivation (body mass and foraging efficiency), neophobia, or weather variables (Table S4). In our main analysis, there was an effect of group size on cognitive performance, whereby individuals from larger groups perform better (Table 2, see Table S5 for full model output). Cognitive performance was also predicted by an interaction between group size and age, whereby individuals raised in larger groups performed better on an associative learning task at 300 days post‐fledgling (Figure 3, Table 2, see Table S5 for full model output). This relationship between group size and cognitive performance was not identified at other ages, complementing the results of previous research on this population (however, this effect was detected at 200 and 300 days post‐fledgling in (Ashton, Ridley, et al. 2018)). For the subset of individuals with social network measures, we found that social network metrics were consistently better predictors than group size (Table S6). Specifically, we found the interactions between vocal in‐degree and age and agonistic in‐degree and age were the best predictors of associative learning performance (Table 3). Fledglings responding to the vocalisations from more conspecifics performed better at the associative learning task at 300 days post‐fledging, but not at the younger ages (Figure 4b, Table 3, see Table S6 for full model output). Additionally, fledglings receiving the most aggression from more conspecifics performed better at the associative learning task at 200‐ and 300‐day post‐fledgling (Figure 4a, Table 3, see Table S6 for full model output). Aggression was not correlated with any of our motivational proxies (VIF < 2, Table S7).

**TABLE 2:** Top model set of candidate terms affecting performance in the associative learning task (*N* = 74 tests on 34 individuals from 10 groups).

	AICc	△AICc
Null model	437.29	6.47
Top model		
Group size	430.82	0
Group size * Age	431.04	0.22

Effect sizes	Effect	SE	95% CI
Intercept	2.67	0.04	2.60/2.74
Group size	−0.14	0.04	−0.21/−0.07
Age	−0.03	0.04	−0.09/0.04
Group size * Age (300 days)	−0.08	0.04	−0.15/−0.00

*Note:* AICc are provided for models within 2 △AICc of the top model and with predictors whose 95% confidence intervals do not intersect 0. Coefficient estimates (Effect), standard error (SE) and 95% confidence intervals (CI) are given below the top model set. Individual identity was included as a random term. See Table S5 for full model selection output.

**TABLE 3:** Top model set of candidate terms affecting performance in the associative task for individuals with social network metrics from the combined testing periods (i.e., scores for all ages were combined to include all cognitive performance data, across all ages, in a single analysis (*N* = 46 tests on 21 individuals from 8 groups)).

	AICc	△AICc
Null model	273.00	4.57
Top model		
Agonistic in‐degree * Age	268.43	0
Vocal in‐degree * Age	269.45	1.02

Effect sizes	Effect	SE	95% CI
Intercept	2.70	0.04	2.62/2.79
Agonistic in‐degree	−0.06	0.04	−0.14/0.02
Age	−0.05	0.04	−0.13/0.03
Vocal in‐degree	−0.00	0.04	−0.08/0.08
Agonistic in‐degree * Age (300 days)	−0.14	0.04	−0.23/−0.06
Vocal in‐degree * Age (300 days)	−0.15	0.05	−0.24/−0.06

*Note:* AICc are provided for models within 2 △AICc of the top model and with predictors whose 95% confidence intervals do not intersect 0. Coefficient estimates (Effect), standard error (SE) and 95% confidence intervals (CI) are given below the top model set. Individual identity was included as a random term. See Table S6 for full model selection output.

## 4 Discussion

We quantified the social connectedness of an individual using a range of social network measures and group size, with the aim of exploring whether the informational challenges associated with the social environment during early life influences cognitive development. We found that cognitive performance is predicted by group size at 300 days post‐fledging, but not at 100 or 200 days, which supports a developmental effect of group size on cognitive development and is consistent with previous research on this species (Ashton, Ridley, et al. 2018; Speechley, Ashton, Thornton, Simmons, et al. 2024). However, we also found that social network metrics are better predictors of cognitive performance than group size. Specifically, we found that fledglings responding to vocalisations from a greater number of conspecifics (vocal in‐degree) and those receiving aggression from more conspecifics (agonistic in‐degree) had higher cognitive performance at 300 days post‐fledging.

Our analysis revealed that although group size is related to cognitive performance at 300 days post‐fledging in magpies, social network measures were consistently better predictors of cognitive performance (see Table S6 for the difference in AICc between social network measures and group size). Additionally, we find that group size and individual social network metrics represent different components of the social environment (indicated by a low correlation between measures of social interaction and group size). Although group size is a valuable measure of sociality, providing the largest potential number of conspecifics with which an individual can interact, it assumes that all individuals within the same group experience the same social pressures, when realistically, individuals in social groups may not interact equally (Bergman and Beehner 2015). For example, a study on mountain gorillas ( *Gorilla beringei beringei* ) found that the number and diversity of relationships varied greatly between individuals within the same group (Morrison et al. 2020), and recent research revealed that the same is true for magpies in our study population (Speechley, Ashton, Thornton, et al. 2024). Specifically, Speechley, Ashton, Thornton, et al. (2024) found that individual cognitive performance of adults was also influenced by individual social interactions beyond total group size. Our current finding that several different social interactions predict cognitive performance in magpie fledglings supports the premise that sociality presents several cognitive challenges, such as needing to recognise and respond to a variety of social situations.

Our study represents one of only a few studies that have incorporated multiple measures of social interactions when investigating the relationship between the social environment and cognitive development. We quantified four types of social interaction and find that responding to vocalisations from a greater number of conspecifics (vocal in‐degree) and receiving aggression from more conspecifics (agonistic in‐degree) predicts an individual's cognitive performance during development. The handful of other studies that have explored the relationship between multiple social network metrics and cognitive performance have also found that specific social interactions are associated with performance in specific cognitive tasks (Ash et al. 2020; Wascher 2015). For instance, in carrion crows ( *Corvus corone corone* ) individuals that initiated more affiliative interactions performed better in quantitative choice and heterospecific recognition tasks, whereas those performing better in inequity aversion initiated fewer affiliative interactions (Wascher 2015). Our results support these findings and suggest that the cognitive challenges generated by the social environment arise from specific types of social interactions.

Our results reveal a relationship between vocal interactions and cognitive development in fledglings, whereby fledglings responding to vocalisations from a greater number of conspecifics have higher cognitive performance at 300 days post‐fledgling, but not at younger ages. This supports a link between sociality and cognitive development, whereby a greater exchange of information via vocalisations is considered cognitively demanding (Freeberg et al. 2012), and that a greater number of vocal interactions is related to cognitive performance. Although vocalisations are increasingly being used in social network studies (Jenikejew et al. 2020; Kulahci et al. 2015), they are rarely related to individual variation in cognitive performance (Ash et al. 2020). Most existing studies compare vocal learning or vocal complexity to cognitive performance in adults (Anderson et al. 2017; Boogert et al. 2011, 2008; Templeton et al. 2014). For example, a study on zebra finches ( *Taeniopygia guttata* ) found a positive association between the total elements per song and performance in a novel foraging problem (Boogert et al. 2008). Additionally, the link between non‐vocal social communication and cognitive performance is an avenue that also requires research (Freeberg et al. 2012). Given the paucity of studies comparing social communication to cognitive performance, our results reveal a rare link between vocal interactions and cognitive development. However, it remains to be determined whether increased vocal interactions lead to improved accuracy when responding to vocalisations.

We also found that individuals who received aggression from more conspecifics performed better in cognitive tasks, which complements recent findings on adults in this species (Speechley, Ashton, Thornton, et al. 2024). Specifically, adult magpies in this study population who received aggressive interactions performed better in associative learning tasks and those involved in aggressive interactions with more group members performed worse (Speechley, Ashton, Thornton, et al. 2024). The relationship between cognition and aggression observed in fledglings may support the “necessity drives innovation” hypothesis, which proposes that individuals will invest more in finding solutions to new problems if they are unable to monopolise resources (Reader and Laland 2003). For example, low‐ranking chimpanzees ( *Pan troglodytes* ) innovate more frequently than high‐ranking chimpanzees (Reader and Laland 2001). However, there is no evidence that aggression is related to resource acquisition in adult magpies, with a food provisioning study finding no relationship between aggression and monopoly of supplemented food (Mitchell 2014), and despite over ten years of observation on the study population, we have not observed kleptoparasitism between group members, suggesting aggression does not facilitate the acquisition of food from others. It is possible that individuals receiving aggression more often are more likely to associate behavioural cues with aggression to avoid future conflict, perhaps to avoid the cost of receiving aggression (e.g., physical injury). Previous work on carrion crows ( *Corvus corone corone* ) hypothesised that aggressive individuals would perform worse in cognitive tests (Wascher 2015). However, their hypothesis was not supported and they found that individuals involved in more pro‐social behaviour performed better in socio‐cognitive tests regardless of the interaction (affiliative or agonistic, Wascher 2015). It is also possible that aggression presents a motivational confound in our study, whereby aggressive individuals are less motivated to complete the test. However, cognitive performance was not predicted by any of our metrics of motivation (body mass or foraging efficiency), nor were they correlated with aggression. Ultimately, it remains feasible that fledglings receiving more aggression perform better in associative learning tasks due to a greater need to learn to associate behavioural cues (such as an aggressive posture or aggressive vocal threat) with incoming aggression and avoid future conflict.

We have presented a detailed exploration of the relationship between social interactions and cognition during the first year of life to determine how cognitive development may be impacted by various measures of sociality, including group size and social network connectivity, based on multiple interaction types. Overall, we found that cognitive development is related to group size, the number of conspecifics an individual vocally responded to, and the number of conspecifics an individual received aggression from. However, it remains to be determined whether the effects of the social environment on cognitive development persist into adulthood. Our findings build on previous research by: (a) showing that social interactions influence cognition at the latter stages of development, confirming that social conditions during development influence cognition, and (b) highlighting the importance of including fine‐grained measures of social interactions, which capture the informational challenges associated with the social environment, specifically in relation to cognitive development.

## Author Contributions

**Elizabeth M. Speechley:** conceptualization (lead), data curation (lead), formal analysis (lead), funding acquisition (supporting), investigation (lead), methodology (equal), resources (supporting), visualization (lead), writing – original draft (lead). **Benjamin J. Ashton:** conceptualization (equal), funding acquisition (equal), methodology (equal), resources (equal), supervision (supporting), writing – review and editing (supporting). **Alex Thornton:** conceptualization (equal), funding acquisition (equal), writing – review and editing (supporting). **Stephanie L. King:** conceptualization (equal), formal analysis (supporting), methodology (supporting), writing – review and editing (supporting). **Leigh W. Simmons:** conceptualization (supporting), investigation (supporting), methodology (supporting), supervision (supporting), writing – review and editing (supporting). **Amanda R. Ridley:** conceptualization (equal), formal analysis (supporting), funding acquisition (equal), investigation (equal), methodology (equal), project administration (equal), resources (equal), supervision (lead), writing – review and editing (equal).

## Conflicts of Interest

The authors declare no conflicts of interest.

## Supporting information


**Data S1:** ece372435‐sup‐0001‐DataS1.xlsx.

**Data S2:** ece372435‐sup‐0002‐DataS2.xlsx.

**Data S3:** ece372435‐sup‐0003‐DataS3.pdf.

**Data S4:** ece372435‐sup‐0004‐DataS4.docx.

## Data Availability

Data available on figshare: https://doi.org/10.6084/m9.figshare.28955582.v1.
