# Supplementary material for: Social Interactions Are Related to Cognitive Development in Western Australian Magpie Fledglings
Source: Ecol Evol. 2025 Nov 11;15(11):e72435. doi: 10.1002/ece3.72435 (PMC12604942; doi:10.1002/ece3.72435)
Supplement: Supplementary file 4 — Data S4: ece372435‐sup‐0004‐DataS4.docx. [file ECE3-15-e72435-s002.docx]

**Supplementary Table 1.** Group based coefficient of variation and permutation p-values for proximity networks cut-offs, restricting interactions to those occurring at distances of 1,2,5 and 15m. Significant p values (<0.05) are in bold.

| **Group** | **Coefficient of Variation** | | | | | | | | | | **Permutations** | | | | | | |
| --- | --- | --- | --- | --- | --- | --- | --- | --- | --- | --- | --- | --- | --- | --- | --- | --- | --- |
|  | **1m** | | **2m** | | **5m** | | **15m** | | **1m** | | | **2m** | | **5m** | | **15m** | |
| BWYa | | 85.39 | | 118.34 | | 104.6 | | 118.35 | | >0.05 | | | 0.2 | | 0.2 | | >0.05 |
| BWYb | | 100.98 | | 104.96 | | 82.71 | | 55.25 | | 0.139 | | | 0.262 | | 0.229 | | 0.542 |
| FMRa | | 61.78 | | 57.03 | | 48.06 | | 44.92 | | 0.235 | | | 0.584 | | **0.028** | | 0.979 |
| JOG | | 106.45 | | 96.12 | | 78.65 | | 64.45 | | **0.03** | | | **0.001** | | 0.087 | | >0.05 |
| KMO | | 95.29 | | 71.04 | | 73.48 | | 74.48 | | >0.05 | | | >0.05 | | >0.05 | | >0.05 |
| LHP | | 159.1 | | 69.15 | | 75.62 | | 67.12 | | >0.05 | | | >0.05 | | >0.05 | | >0.05 |
| MBG | | 82.12 | | 65.52 | | 58.72 | | 59.38 | | 0.113 | | | 0.056 | | 0.3 | | 0.3 |
| NH | | 57.84 | | 49.98 | | 49.74 | | 47.22 | | 0.931 | | | 0.533 | | 0.642 | | 0.997 |
| PR | | 68.37 | | 86.26 | | 79.43 | | 63.97 | | 0.957 | | | 0.912 | | 0.858 | | 0.712 |
| RVD | | 86.17 | | 96.36 | | 71.37 | | 63.67 | | **0.021** | | | 0.636 | | 0.267 | | 0.866 |
| SCL | | 116.35 | | 118.59 | | 75.87 | | 82.09 | | >0.05 | | | >0.05 | | >0.05 | | >0.05 |
| SS | | 84.36 | | 103.78 | | 73.15 | | 92.64 | | 0.469 | | | 0.987 | | 0.912 | | 0.639 |


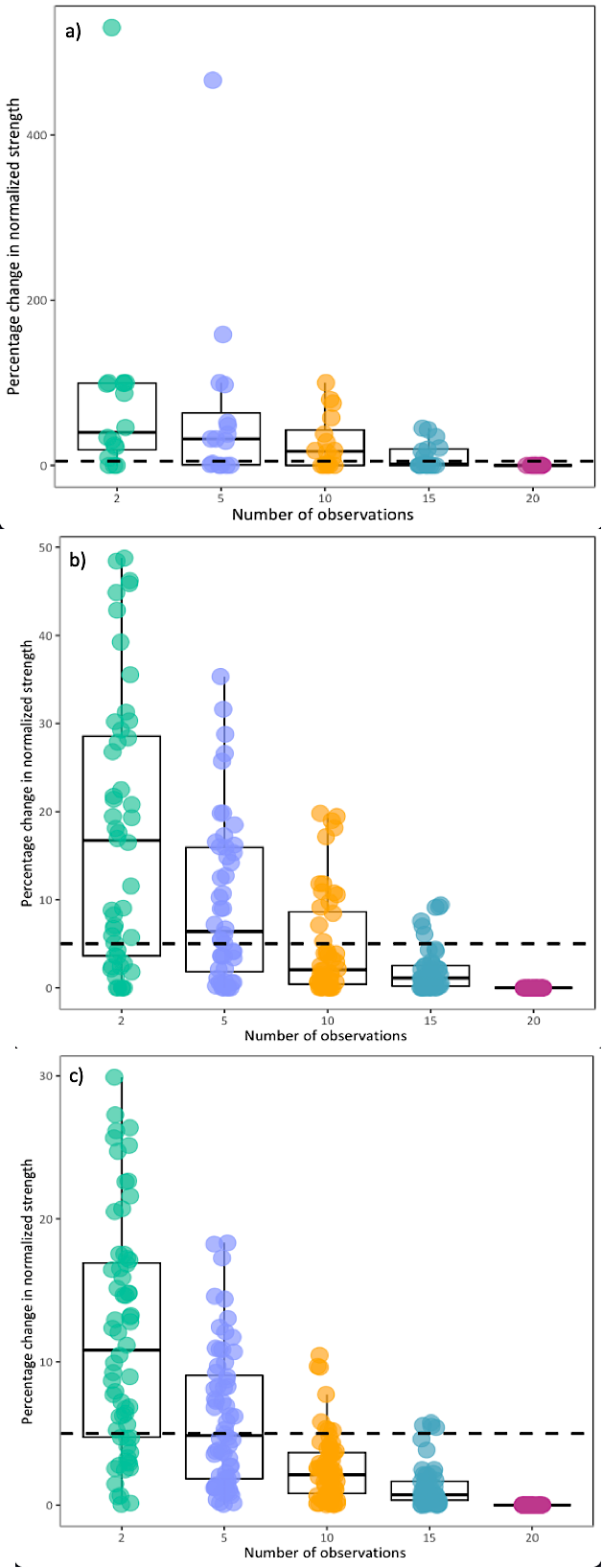


Percentage change in normalised strength

Percentage change in normalised strength

Percentage change in normalised strength

**Supplementary Figure 1.** Sensitivity analysis conducted on 2, 5, 10, 15 and 20 observations per focal individual in the a) play (n=5 groups), b) agonistic (n=11 groups) and c) vocal networks (n=11 groups). For each observation number, the number of observations was randomly selected from the full dataset to calculate normalised strength for each focal 1000 times, thus giving an average normalised strength for each individual at each observation number. Percentage change in normalised strength was then calculated for each individual and observation number and compared to the full dataset. The horizontal line represents a 5% change in normalised strength (an arbitrary cut-off).

**Supplementary Table 2.** The number of fledglings tested out of the total number of fledglings in the group for each testing period (100, 200 and 300 days post-fledgling) for 2020 and 2021.

| **Group** | **2020** | | | **2021** | | |
| --- | --- | --- | --- | --- | --- | --- |
|  | **100** | **200** | **300** | **100** | **200** | **300** |
| BWYb | 2/2 | 2/2 | 2/2 | 2/2 | 2/2 | 2/2 |
| CPC | 2/2 | 2/2 | 2/2 | NA | NA | NA |
| ESC | 1/3 | 2/3 | 2/3 | 2/3 | 2/3 | 2/3 |
| FMRa | 0/2 | 1/2 | 1/2 | 1/1 | 0/0 | 0/0 |
| JOG | 5/5 | 2/2 | 1/1 | 4/4 | 3/4 | 1/1 |
| LHP | NA | NA | NA | 2/2 | 2/2 | 0/0 |
| MBG | 2/2 | 2/2 | 1/2 | 1/2 | 2/2 | 0/0 |
| NH | NA | NA | NA | 0/4 | 2/4 | 0/3 |
| RVD | 3/3 | 3/3 | 3/3 | NA | NA | NA |
| SCL | NA | NA | NA | 1/1 | 1/1 | 1/1 |

**Supplementary Table 3.** Permutation p-values for each group during each testing period based on four different interaction types (affiliative, agonistic, vocal and proximity). Significant p values (<0.05) are in bold.

| **Group** | **Year** | **Time period** | **Permutation p-value** | | | |
| --- | --- | --- | --- | --- | --- | --- |
|  |  |  | **Affiliative** | **Agonistic** | **Vocal** | **Proximity** |
| BWYa | 2020 | February to June | NA | >0.05 | **<0.001** | >0.05 |
| BWYb | 2020 |  | **<0.001** | **<0.001** | **<0.001** | 0.139 |
| FMRa | 2020 |  | 0.105 | **<0.001** | **<0.001** | 0.235 |
| JOG | 2020 |  | **<0.001** | 0.057 | **<0.001** | **0.03** |
| KMO | 2020 |  | NA | NA | **<0.001** | >0.05 |
| LHP | 2020 |  | NA | NA | **<0.001** | >0.05 |
| MBG | 2020 |  | **<0.001** | **0.01** | **<0.001** | 0.113 |
| NH | 2020 |  | NA | >0.05 | **<0.001** | 0.931 |
| PR | 2020 |  | NA | **<0.001** | **<0.001** | 0.957 |
| RVD | 2020 |  | **<0.001** | **<0.001** | **<0.001** | **0.021** |
| SCL | 2020 |  | NA | NA | **<0.001** | >0.05 |
| SS | 2020 |  | NA | NA | **<0.001** | 0.469 |
| BWYa | 2020 | August to October | NA | >0.05 | 0.871 | >0.05 |
| BWYb | 2020 |  | **<0.001** | **<0.001** | **<0.001** | 0.106 |
| CPC | 2020 |  | **<0.001** | **0.028** | **<0.001** | >0.05 |
| FMRa | 2020 |  | **<0.001** | **<0.001** | **<0.001** | **<0.001** |
| JOG | 2020 |  | **<0.001** | **<0.001** | **<0.001** | 0.539 |
| KMO | 2020 |  | NA | >0.05 | 0.113 | >0.05 |
| LHP | 2020 |  | NA | NA | >0.05 | >0.05 |
| MBG | 2020 |  | **0.006** | **0.016** | **<0.001** | 0.541 |
| NH | 2020 |  | NA | 0.343 | **<0.001** | 0.625 |
| PR | 2020 |  | >0.05 | **<0.001** | **<0.001** | 0.578 |
| RVD | 2020 |  | **<0.001** | **<0.001** | **<0.001** | >0.05 |
| SCL | 2020 |  | NA | NA | **0.014** | >0.05 |
| SS | 2020 |  | **<0.001** | **<0.001** | **<0.001** | 0.975 |
| BWYa | 2021 | February to June | NA | NA | **<0.001** | NA |
| BWYb | 2021 |  | **<0.001** | **0.014** | **<0.001** | 0.405 |
| CPC | 2021 |  | **0.022** | **0.018** | **<0.001** | 0.699 |
| FMRa | 2021 |  | >0.05 | **<0.001** | **<0.001** | **0.011** |
| JOG | 2021 |  | **0.004** | **<0.001** | **<0.001** | 0.588 |
| KMO | 2021 |  | NA | NA | **<0.001** | 0.2 |
| LHP | 2021 |  | **<0.001** | **<0.001** | **<0.001** | **0.009** |
| MBG | 2021 |  | **<0.001** | **<0.001** | **<0.001** | 0.3107 |
| NH | 2021 |  | 0.121 | **<0.001** | **<0.001** | 0.577 |
| PR | 2021 |  | NA | 0.417 | **<0.001** | 0.303 |
| RVD | 2021 |  | **0.023** | **0.005** | **<0.001** | 0.6 |
| SCL | 2021 |  | **0.009** | 0.188 | **<0.001** | 0.2 |
| SS | 2021 |  | NA | 0.656 | **<0.001** | 0.301 |
| BWYa | 2021 | August to October | NA | NA | >0.05 | NA |
| BWYb | 2021 |  | **<0.001** | **<0.001** | **<0.001** | 0.226 |
| CPC | 2021 |  | NA | 0.587 | 0.113 | 0.545 |
| FMRa | 2021 |  | NA | **0.013** | **0.031** | 0.461 |
| JOG | 2021 |  | **<0.001** | **0.013** | **<0.001** | 0.251 |
| KMO | 2021 |  | NA | NA | 0.056 | 0.1 |
| LHP | 2021 |  | **0.035** | 0.169 | **<0.001** | 0.202 |
| MBG | 2021 |  | >0.05 | **0.037** | **<0.001** | 0.485 |
| NH | 2021 |  | **<0.001** | **<0.001** | **<0.001** | 0.408 |
| PR | 2021 |  | NA | 0.565 | **<0.001** | 0.1 |
| RVD | 2021 |  | NA | **<0.001** | **<0.001** | 0.488 |
| SCL | 2021 |  | >0.05 | 0.213 | **<0.001** | NA |
| SS | 2021 |  | NA | **<0.001** | **<0.001** | 0.3 |

The exploratory analysis below is designed to explore potential confounding factors that may influence cognitive performance such as motivational or environmental factors. We conduct this analysis on the full data set prior to the main analysis. Also note that when testing we also recorded any anti-predator behaviours (e.g. increased vigilance and alarm calls) that occurred during testing in case they impacted individual performance. However, there were not enough instances of anti-predator behaviour to analyse so it has not been included in analyses.

**Supplementary Table 4.** Full model set of additional terms that may inadvertently affect cognitive performance in the associative learning test (N=74 tests on 34 individuals from 10 groups using model selection from the combined testing periods. Individual identity was included as a random term. Terms in top model set with confidence intervals not intersecting 0 are in bold. Corrected AICc and △AICc are provided for all models. See Supplementary Table 2 for a glossary of terms.

| **Terms** | **AICc** | **△AICc** |
| --- | --- | --- |
| Shade | 436.57 | 0 |
| Null | 437.29 | 0.72 |
| Body Mass | 438.32 | 1.75 |
| Temperature | 438.95 | 2.38 |
| Foraging Efficiency | 439.13 | 2.56 |
| Order Tested | 439.15 | 2.58 |
| Neophobia | 439.49 | 2.92 |

**Supplementary Table 5.** Full model set of candidate terms affecting fledgling performance in the associative learning test (N=74 tests on 34 individuals from 10 groups using model selection from the combined testing periods. Individual identity was included as a random term. Terms in top model set with confidence intervals not intersecting 0 are in bold. Corrected AICc and △AICc are provided for all models. See Supplementary Table 2 for a glossary of terms.

| **Terms** | **AICc** | **△AICc** |
| --- | --- | --- |
| Group Size | 430.82 | 0 |
| Group size * Age | 431.04 | 0.22 |
| Null | 437.29 | 6.47 |
| Brood Mates | 438.49 | 7.67 |
| Number of Fledglings | 438.68 | 7.86 |
| Age | 439.45 | 8.62 |

**Supplementary Table 6.** Full model set of candidate terms affecting performance in the associative learning test using model selection for individuals with social network metrics (N=46 tests on 21 individuals from 8 groups). Individual identity was included as a random term. Terms in top model set with confidence intervals not intersecting 0 are in bold. Corrected AICc and △AICc are provided for all models. COV represents coefficient of variation. See Supplementary Table 2 for a glossary of terms.

| **Terms** | **AICc** | | **△AICc** |
| --- | --- | --- | --- |
| Agonistic In-Degree * Age | 268.43 | | 0 |
| Vocal In-Degree * Age | 269.45 | | 1.02 |
| Affiliative Strength | 271.00 | | 2.57 |
| Group Size | 271.25 | | 2.82 |
| Affiliative Strength * Age | 271.63 | | 3.21 |
| Null | 273.00 | | 4.57 |
| Brood Mates | 273.14 | | 4.72 |
| Agonistic In-Strength * Age | 273.21 | | 4.79 |
| Age | 273.84 | | 5.42 |
| Affiliative COV | 274.14 | | 5.71 |
| Agonistic Out-Strength | 274.20 | 5.78 | |
| Agonistic In-Strength | 274.62 | 6.19 | |
| Vocal COV | 274.70 | 6.28 | |
| Agonistic In-Degree | 274.78 | 6.35 | |
| Vocal In-Degree | 275.00 | 6.58 | |
| Vocal Out-Degree | 275.09 | 6.67 | |
| Number of Fledglings | 275.16 | 6.73 | |
| Group Size * Age | 275.17 | 6.74 | |
| Vocal Out-Strength | 275.39 | 6.96 | |
| Vocal In-Strength | 275.39 | 6.97 | |
| Agonistic Out-Degree | 275.40 | 6.97 | |
| Agonistic COV | 275.40 | 6.98 | |
| Vocal In-Strength * Age | 276.21 | 7.78 | |
| Agonistic Out-Degree * Age | 276.69 | 8.26 | |
| Agonistic Out-Strength * Age | 276.92 | 8.49 | |
| Vocal COV * Age | 277.55 | 9.12 | |
| Vocal Out-Strength * Age | 277.83 | 9.41 | |
| Agonistic COV * Age | 277.90 | 9.47 | |
| Vocal Out-Degree * Age | 278.24 | 9.82 | |

**Supplementary Table 7.** Variance inflation factor (VIF) values for correlations between agonistic social network metrics and proxies of motivation (body mass and foraging efficiency). VIF values <2 indicates low level correlation.

|  | **Agonistic degree** | | **Agonistic in-degree** | **Agonistic out-degree** | **Agonistic strength** | **Agonistic in-strength** | **Agonistic out-strength** | **Agonistic COV** |
| --- | --- | --- | --- | --- | --- | --- | --- | --- |
| **Body mass** | | 1.03 | 1.11 | 1.00 | 1.03 | 1.17 | 1.07 | 1.00 |
| **Foraging efficiency** | | 1.08 | 1.02 | 1.02 | 1.09 | 1.00 | 1.05 | 1.02 |
